# Sitting and standing performance in a total population of children with cerebral palsy: a cross-sectional study

**DOI:** 10.1186/1471-2474-11-131

**Published:** 2010-06-23

**Authors:** Elisabet Rodby-Bousquet, Gunnar Hägglund

**Affiliations:** 1 Department of Orthopaedics, Lund University, University Hospital, Lund, Sweden; 2 Centre for Clinical Research, Uppsala University, Central Hospital, Västerås, Sweden

## Abstract

**Background:**

Knowledge of sitting and standing performance in a total population of children with cerebral palsy (CP) is of interest for health care planning and for prediction of future ability in the individual child. In 1994, a register and a health care programme for children with CP in southern Sweden was initiated. In the programme information on how the child usually sits, stands, stands up and sits down, together with use of support or assistive devices, is recorded annually.

**Methods:**

A cross-sectional study was performed, analysing the most recent report of all children with CP born 1990-2005 and living in southern Sweden during 2008. All 562 children (326 boys, 236 girls) aged 3-18 years were included in the study. The degree of independence, use of support or assistive devices to sit, stand, stand up and sit down was analysed in relation to the Gross Motor Function Classification System (GMFCS), CP subtype and age.

**Result:**

A majority of the children used standard chairs (57%), could stand independently (62%) and could stand up (62%) and sit down (63%) without external support. Adaptive seating was used by 42%, external support to stand was used by 31%, to stand up by 19%, and to sit down by 18%. The use of adaptive seating and assistive devices increased with GMFCS levels (p < 0.001) and there was a difference between CP subtypes (p < 0.001). The use of support was more frequent in preschool children aged 3-6 (p < 0.001).

**Conclusion:**

About 60% of children with CP, aged 3-18, use standard chairs, stand, stand up, and sit down without external support. Adding those using adaptive seating and external support, 99% of the children could sit, 96% could stand and 81% could stand up from a sitting position and 81% could sit down from a standing position. The GMFCS classification system is a good predictor of sitting and standing performance.

## Background

Cerebral palsy (CP) is the most frequent cause of motor disability in children and adolescents [1-3] with a prevalence of 2-3 children per 1000 live births [2,4]. The severity of impairments is extremely variable. Almost one third of children with CP are non-ambulant and spend most of their lives in a sitting or lying position [3]. Reduced postural ability is often a key problem and they may need postural support or assistive devices to stabilize the body against gravity in order to maintain a sitting or standing position [5-16]. A crouched standing posture leads to a reduced hip and knee extension that worsens over time, due to gravity and the altered position of the body segments in relation to each other [17]. Postural control is also affected by the environment and the attention required when performing dual tasks [18]. Adaptive seating reduces the need for assistance from a caregiver [19-21] and may facilitate daily activities and functions such as playing [22], eating [20-24], breathing [25] and arm and hand function [8,26,27].

According to the Surveillance of Cerebral Palsy in Europe network (SCPE) [2] the CP subtypes are classified as Spastic Unilateral, Spastic Bilateral, Ataxic, Dyskinetic and Unclassified or mixed types. To define the level of gross motor function in children with CP, the Gross Motor Function Classification System (GMFCS) [28,29] is generally accepted.

A strong predictor for ambulation in children with CP is sitting ability at two years of age [30,31]. Knowledge of the sitting and standing performance in a total population of children with CP is of interest for health care planning and to predict future ability in the individual child.

In 1994, a CP register and a health care programme, called CPUP, was initiated for children with CP in southern Sweden [32,33]. The child's local physiotherapist fills in a recording form twice a year up to six years of age and once a year thereafter. These data have been used to analyse sitting and standing performance in a total population of children with CP.

### Purpose

To describe how children with CP usually sit, stand, stand up and sit down and their use of support/assistive devices, related to age, CP subtype and GMFCS level.

## Methods

The CPUP register includes all children with CP born after 1 January 1990 living in the counties of Skåne and Blekinge in southern Sweden, which have a total population of about 1.3 million. The number of children with CP in the area corresponds to a prevalence of 2.4 per 1000 live births [4,34]. Since 2005, CPUP has been a National Health Care Quality Register approved by the National Board of Health and Welfare in Sweden. A search is made regularly to find all children with CP in the area and invite them to participate in CPUP [4]. Almost all families (98%) have agreed to participate [4].

In the present study the most recent reports in all children with CP born 1990-2005 and living in the area in 2008 were analysed. There were 562 children in total (326 boys, 236 girls) aged 3-18 years (mean age 10.9) in the register and they were all included in the study. The distribution of GMFCS levels was: level I 47.1%, II 13.5%, III 11.4%, IV 15.0% and V 13.0%. For the subtypes the distribution was: spastic unilateral 29%, spastic bilateral 37.2%, ataxic 8.5%, dyskinetic 14.8% and unclassified or mixed types 10.5%. For more detailed distribution of age, sex, GMFCS level and subtype, see Table 1.

**Table 1:** Details of the 562 children, their age, sex, GMFCS level and CP subtype.

Age	No. Of children	Sex		GMFCS level					CP subtype				
		B	G	I	II	III	IV	V	S U	S B	DY	AT	UC
**3**	25	12	13	10	3	3	5	4	3	3	2	1	16
**4**	27	16	11	16	2	2	5	2	6	4	6	2	9
**5**	32	18	14	16	1	7	2	6	8	9	5	3	7
**6**	32	21	11	9	3	9	4	7	8	11	8	1	4

**7**	29	13	16	15	6	3	3	2	9	11	3	2	4
**8**	40	22	18	19	3	3	10	5	12	16	5	5	2
**9**	35	18	17	18	5	2	6	4	14	11	5	2	3

**10**	40	27	13	12	10	6	8	4	12	19	7	0	2
**11**	33	15	18	14	4	6	5	4	8	13	5	3	4
**12**	44	25	19	25	8	2	4	5	18	15	5	2	4

**13**	34	19	15	19	3	3	4	5	12	16	4	2	0
**14**	34	23	11	19	6	1	4	4	10	15	2	5	2
**15**	49	36	13	21	5	3	10	10	15	18	10	6	0

**16**	37	21	16	19	6	3	6	3	11	15	5	5	1
**17**	40	23	17	20	7	6	6	1	8	18	6	7	1
**18**	31	17	14	12	4	5	2	8	9	15	5	2	0

**Total**	562	326	236	264	76	64	84	74	163	209	83	48	59

SU=Spastic unilateral CP, SB=Spastic bilateral CP, DY=Dyskinetic CP, AT=Ataxic CP, UC=Unclassified or mixed type

The programme includes a continuous standardized follow-up of gross and fine motor function, clinical findings and treatment. The child's local physiotherapist examines the child and fills in a recording form twice a year until the age of six, then once a year. The recording form includes information about how the child usually sits, stands, stands up and sits down. The information about the child's performance (what they usually do) is obtained by questions put to the children and their caregivers.

The degree of independence and use of support or assistive devices to sit, stand, stand up and sit down were analysed in relation to subtype, GMFCS level and age. The CP subtype for each child was determined by the child's neuropaediatrician and the GMFCS level by its local physiotherapist. To analyse differences in data at different ages the children were divided into five age groups according to the Swedish school system, 3-6, 7-9, 10-12, 13-15 and 16-18 years.

The International Classification of Functioning, Disability and Health (ICF) [35] was used to define the different activities as follows: to sit = maintain a basic body position in sitting; to stand = maintain a basic body position in standing; to stand up = change a basic body position and get into a standing position from a seated position; to sit down = change a basic body position and get into a seated position, sit down (on a chair) from a standing position.

The question regarding seating was: What kind of chair does the child usually sit in? The options were: The child uses (1) a standard chair, (2) adaptive seating or (3) does not sit. Adaptive seating was defined as any special seating, high chair or seating system provided as an assistive device to those who cannot sit in a standard chair due to postural deficit and/or physical disability.

The questions regarding standing were: How does the child usually get into a standing position from sitting on a chair; maintain a standing position; sit down on a chair from a standing position? The options were: The child (1) does it independently without external support; (2) does it with external support or (3) cannot. External support denotes support from the environment (wall, furniture, assistive devices) or from another person.

SPSS version 17.0 was used for the statistical analyses. The Kruskal-Wallis test was used to analyse differences related to CP subtypes. Mann-Whitney was used for post hoc tests for Kruskal-Wallis. Linear by linear association test was used to analyse differences related to GMFCS levels and age groups. Spearman's rank correlation was used to calculate correlations for ordinal data. P-values < 0.05 were considered significant.

The study was approved by the Medical Research Ethics Committee at Lund University (LU-443-99).

## Results

### Sitting

**Figure 1:**
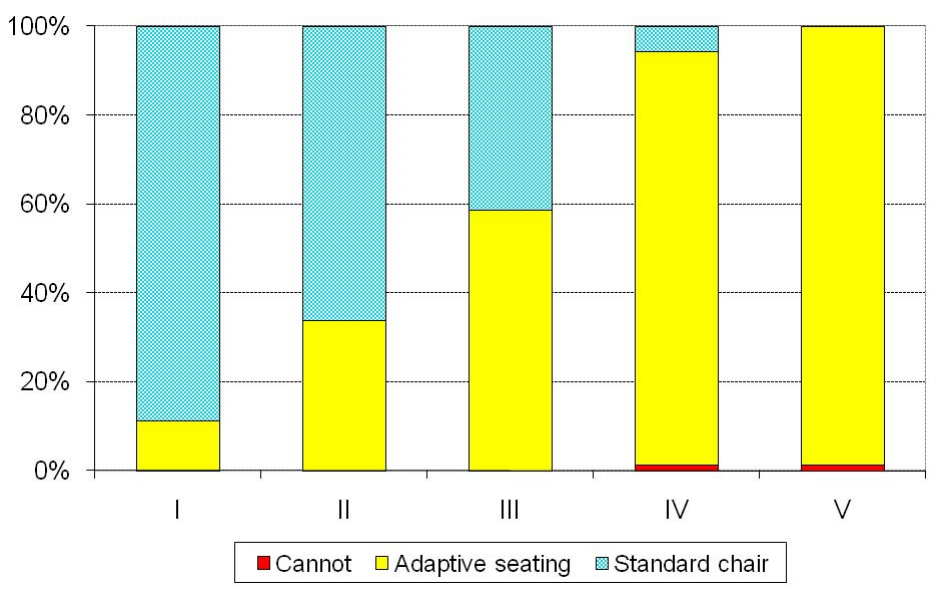
**Type of chair used for sitting related to GMFCS level**. Information missing in 3 of the 562 children aged 3-18 years. The use of adaptive seating increased with GMFCS-levels (p < 0.001), linear by linear association test.

**Figure 2:**
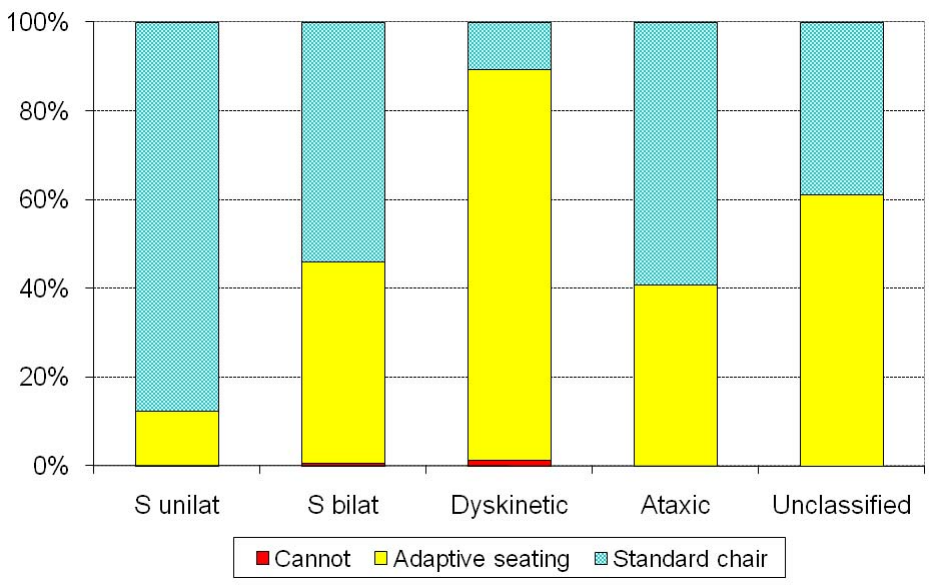
**Type of chair used for sitting related to CP subtype**. Information missing in 3 of the 562 children aged 3-18 years. Kruskal-Wallis test showed a significant difference in use of standard chairs between CP-subtypes (p < 0.001) Post hoc analyses with Mann-Whitney showed a significant difference between all subtypes (p < 0.001) except for ataxic/spastic bilateral, ataxic/unclassified and spastic bilateral/unclassified.

Of the 562 children, 321 (57%) used standard chairs and 236 (42%) used adaptive seating. Two children did not sit, and information was missing in three children. There was a significant increase in use of standard chairs with age (p < 0.001) (Table 2). The use of chairs correlated to GMFCS level (r_s _= 0.73, p < 0.001) (Table 3) and the use of adaptive seating increased with GMFCS levels (p < 0.001). Of the children at GMFCS level I, 90% use standard chairs, 68% at level II, 44% at level III, 5% at level IV and none of the children at level V (Figure 1). Kruskal-Wallis test showed a significant difference in the use of standard chairs between CP subtypes (p < 0.001). Post hoc analyses with Mann-Whitney showed a difference between all subtypes (p < 0.001) except for ataxic/spastic bilateral, ataxic/unclassified and spastic bilateral/unclassified. The use of standard chairs was most frequent in children with spastic unilateral CP (88%), followed by 60% of those with ataxic CP, 54% with spastic bilateral CP and least frequent in children with dyskinetic CP (11%). The reverse was seen for adaptive seating (Figure 2).

**Table 2:** Distribution (%) of use of support (adaptive seating, standing device, external support) to sit, stand, stand up and sit down in different age groups.

	Age group (years)	Without support %	With support %	Cannot %
**Sit**	3-6	40.5	58.6	0.2
	7-9	49.6	40.4	0.0
	10-12	55.6	43.6	0.0
	13-15	59.8	38.5	0.0
	16-18	71.3	27.8	0.2

**Stand**	3-6	55.2	42.2	2.6
	7-9	67.0	30.1	2.9
	10-12	70.1	27.4	2.6
	13-15	65.8	27.4	6.8
	16-18	70.4	25.9	3.7

**Stand up**	3-6	51.7	23.3	25.0
	7-9	66.3	17.3	16.3
	10-12	65.0	21.4	13.7
	13-15	63.8	17.2	19.0
	16-18	65.7	17.6	16.7

**Sit down**	3-6	53.0	17.4	29.6
	7-9	66.3	16.3	17.3
	10-12	65.8	20.5	13.7
	13-15	63.2	17.1	19.7
	16-18	66.7	16.7	16.7

Linear by linear association test showed a significant increase in use of standard chairs with age (p < 0.001). There was a difference in performance to stand (p < 0.05) and to stand up (p = 0.03) between the preschool children 3--6 years compared to the other age groups. The use of support to sit down from standing decreased with age (p = 0.041).

**Table 3:** Correlations between GMFCS levels and independence/use of support to sit, stand, stand up and sit down.

	GMFCS	Sit	Stand	Stand up	Sit down
**GMFCS**	1.00	0.73	0.85	0.88	0.88
**Sit**	0.73	1.00	0.70	0.72	0.72
**Stand**	0.85	0.70	1.00	0.91	0.91
**Stand up**	0.88	0.72	0.91	1.00	0.99
**Sit down**	0.88	0.72	0.91	0.99	1.00

All correlations are statistically significant (p < 0.001) (Spearman's correlation).

### Standing

**Figure 3:**
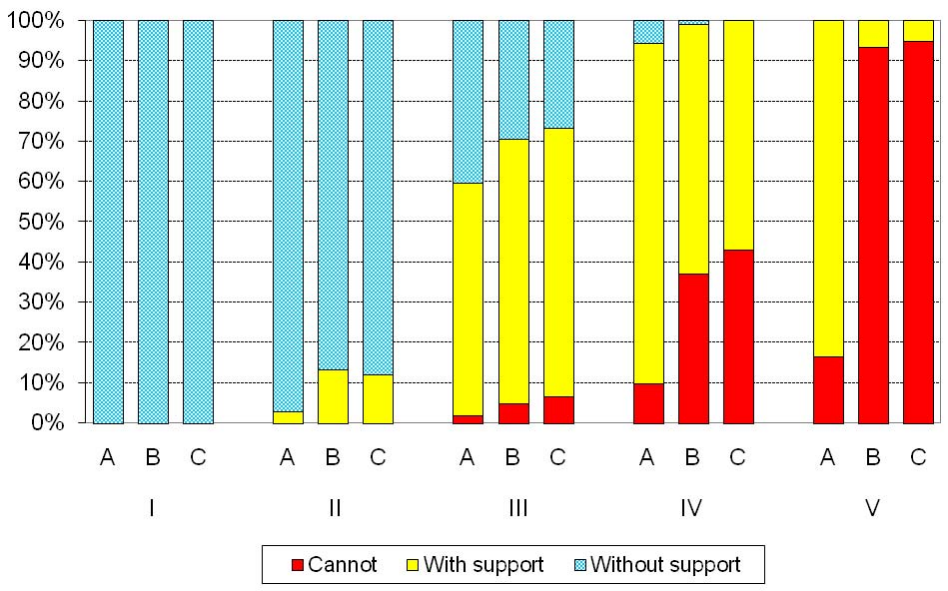
**The use of support to stand (A), stand up (B) and sit down (C) related to GMFCS level**. Information missing in one of the 562 children aged 3-18 years. The use of support increased with GMFCS level (p < 0.001), linear by linear association test.

**Figure 4:**
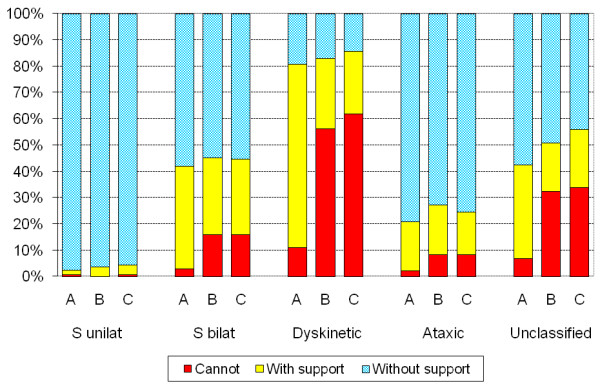
**The use of support to stand (A), stand up (B) and sit down (C) related to CP subtype**. Information missing in one of the 562 children aged 3-18 years. Kruskal-Wallis test showed a significant difference between CP subtypes (p < 0.001) to stand, stand up and sit down. Post hoc analyses with Mann-Whitney revealed a difference between all subtypes (p < 0.05) except for spastic bilateral vs. unclassified.

The reported means of standing showed that 368 (65%) of the children could stand independently without support while 172 (31%) stood with support/assistive devices and 21 (4%) could not stand. Information was missing in one child. The most frequent standing device was a standing brace used by 130 children (75%) in some cases used in combination with a standing frame or a tilt table. Standing frames or tilt tables were used by 57 children, and standing wheelchairs by 23 children. The use of support to stand correlated to the use of adaptive seating (r_s _= 0.70, p < 0.001) and to GMFCS level (r_s _= 0.85, p < 0.001) (Table 3). Standing without support increased with decreasing GMFCS level (p < 0.001). At GMFCS level I all children stood without support while less than half (37%) did so at GMFCS level III and none at level V. However, 84% of all children at GMFCS levels IV-V stood with support/assistive devices (Figure 3). There was a significant difference in standing performance between CP subtypes (p < 0.001). Post hoc analyses showed a significant difference between all subtypes (p < 0.02) except for spastic bilateral vs. unclassified. Of the children with spastic unilateral CP, 98% stood independently, 79% of those with ataxia and 58% of those with spastic bilateral CP, while only 19% of the dyskinetic subtype did so. Support was most frequently used by children with the spastic bilateral and dyskinetic subtypes, in total 48% (Figure 4). There was a difference in standing performance between the preschool children and the other age groups (p < 0.05) (Table 2).

### Standing up from a sitting position

Of the 562 children 350 (62%) could stand up without external support, 109 (19%) required external support and 102 (18%) could not stand up from a sitting position. Information was missing in one child. The use of support to stand up from sitting increased with GMFCS level (p < 0.001). All children at GMFCS level I and 87% at level II got into standing position independently. At GMFCS levels III and IV almost two thirds (64%) required support and at GMFCS level V only 7% got into standing position with support (Figure 3). The use of support to stand up was closely correlated to the use of support to sit down (r_s _= 0.99, p < 0.001) and to GMFCS level (r_s _= 0.88, p < 0.001) (Table 3). There was a significant difference between CP subtypes (p < 0.001). Post hoc analyses showed a significant difference between all subtypes (p < 0.03) except for spastic bilateral vs. unclassified. All children with spastic unilateral CP could get into standing position and only 4% required support. Of the children with ataxic CP, 73% got into standing position without support and 19% used support, while 55% of those with spastic bilateral CP got into standing position without support, 29% used support and 16% could not stand up from sitting. In the dyskinetic subtype only 17% got into standing position independently while 55% could not stand up even with support (Figure 4). More children got into standing position without support in the age groups 7-18 years than those aged 3-6 years (p = 0.03) (Table 2).

### Sitting down from a standing position

The use of support to sit down from standing was similar to that found for getting into standing position (r_s _= 0.99, p < 0.001) (Table 2, 3). Of the 562 children 353 (62%) sat down from standing without support, 99 (18%) used support and 109 (19%) could not. Information was missing in one child. The use of support to sit down decreased with increasing age (Table 2) and increased with GMFCS level (p < 0.001) (Figure 3). All children at GMFCS level I sat down independently and 89% of those at level II. At GMFCS level III, 61% used support to sit down and 57% at level IV. At GMFCS level V only 5% sat down using support and 95% could not (Figure 3). Kruskal-Wallis test showed a significant difference in the way the children sat down from standing between the subtypes (p < 0.001). Post hoc analyses showed a significant difference between all subtypes (p < 0.01) except for spastic bilateral vs. unclassified subtype. In the spastic bilateral subtype 28% used support to sit down, 22% of the children with dyskinetic CP and 15% with ataxic CP. A majority of the children with dyskinetic CP (61%) could not sit down even with support. That was also the case for 16% of the children with spastic bilateral CP and for 8% of those with ataxia (Figure 4).

There were 368 children who stood independently, and of those 346 (94%) got into standing position without support. Of the 172 children who stood with support, 85 also required support to get into standing position and 84 could not do so even with support

## Discussion

This study describes sitting and standing performance in a total population of children with CP and the numbers of those needing assistive devices or external support. The results show what the children usually do, i.e. their performance, not what they can do, i.e. their ability. In children with disabilities, ability exceeds performance [36]. The discrepancy between ability and performance can relate to differences in environmental factors [36]. Most of the children with CP use standard chairs (57%) and can stand (66%), stand up (62%), and sit down (63%) without external support. The children in this study were all included in the CPUP programme which has been shown to reduce the number of severe contractures [33] and hip dislocations [32]. This may have reduced the number of children who were unable to sit or stand and may also have affected the use of assistive devices.

In Sweden assistive devices such as adaptive seating and standing devices are provided free of charge by the Assistive Technology Centres. This means that the results of this study reflect the children's use of assistive devices without regard to the economic situation of the family. The opinions of the child and family, the rehab team and the physical surroundings influence the need for, or use of, adaptive seating and assistive devices. Strategies to alter the environment in order to compensate for functional impairment have recently been recognized in paediatric rehabilitation [21,37,38]. Assistive devices have been shown to enhance function in children with CP and reduce the demand on caregivers [21,37]. It is therefore important that different types of assistive device for sitting and standing are carefully considered for children with CP.

Sitting and standing performance was related to the CP subtype. Almost all children with spastic unilateral CP used standard chairs and did not use assistive devices to stand, stand up or sit down, so this subtype predicts a better sitting and standing performance than the other subtypes. Children with the dyskinetic subtype are the least likely to achieve a high sitting and standing performance since less than 20% sit in standard chairs and stand, stand up and sit down without support. Nearly 40% of the children with CP are classified as spastic bilateral, representing children with all levels of gross motor function. For these children the subtype does not give sufficient information regarding the individual child's sitting and standing performance.

The number of children using adaptive seating decreased with age. The use of support to stand, stand up and sit down was more frequent in preschool children and decreased in schoolchildren and adolescents. However it was not clarified whether this was due to natural development or to environmental factors.

The use of adaptive seating and standing devises increased with GMFCS levels. There was a significant difference (p < 0.001) and a high correlation (r_s _= 0.73-0.88) for all outcome measures related to the GMFCS levels. The GMCS level is age-related and as most children remain at the same GMFCS level this classification system seems useful for prediction of the individual child's future sitting and standing performance.

This material represents a total population of children with CP, aged 3 to 18 years, and therefore the study is likely to give a true picture of the sitting and standing performance and the use of support/assistive devices among children with CP. There is a need for further research on how often the children sit and stand and how long they remain in different positions.

## Conclusion

About 60% of children with CP, aged 3-18, use standard chairs, stand, stand up, and sit down without external support. Adding those using adaptive seating and external support, 99% of the children could sit, 96% could stand and 81% could stand up from a sitting position and 81% could sit down from a standing position. Spastic unilateral or ataxic subtype predicts a better sitting and standing performance than the other subtypes. The GMCS level is age-related and as most children remain at the same GMFCS level this classification system seems useful for prediction of the individual child's future sitting and standing performance.

## Abbreviations

CP: Cerebral Palsy; CPUP: National Health Care Quality Register for Cerebral Palsy; GMFCS: Gross Motor Function Classification System; ICF: International Classification of Functioning, disability and health; SCPE: Surveillance of Cerebral Palsy in Europe network.

## Competing interests

The authors declare that they have no competing interests.

## Authors' contributions

ERB and GH designed the study. Both authors analysed the results. ERB wrote the first draft, which was then actively improved and revised by both authors.

## Pre-publication history

The pre-publication history for this paper can be accessed here:

http://www.biomedcentral.com/1471-2474/11/131/prepub
